# Population models for the main indicators of mental health of the Russian population in the period 1992-2022

**DOI:** 10.1192/j.eurpsy.2025.802

**Published:** 2025-08-26

**Authors:** V. Mitikhin, T. Solokhina, D. Oshevsky

**Affiliations:** 1 Mental Health Research Centre, Moscow, Russian Federation

## Abstract

**Introduction:**

The mental health of the population at the level of the psychiatric service has a number of main indicators: the levels of general morbidity (prevalence) and primary morbidity (incidence) of mental disorders. Population models allow you to demonstrate the scale of problems and assess the resources to solve them.

**Objectives:**

Building population-based epidemiological models of mental health of the Russian population to analyze the relationship between indicators of mental disorders and the human resource of psychiatric care, as well as demographic, socio-economic factors in the period 1992-2022.

**Methods:**

The work uses data from Russian socio-economic statistics, materials from medical and research institutions and results published in scientific periodicals (see, [1] and references). In the formation of population models, the methods of systematic data analysis presented in the work [1] and statistical analysis in the framework of MS Excel were used.

**Results:**

At the first stage, correlation analysis was used to select demographic, socio-economic factors and the factor of the personnel resource of psychiatric care, which significantly affect the indicators of general and primary morbidity of mental disorders. It turned out that the most significant factors are: population size, life expectancy at birth, unemployment rate, number of psychiatrists and psychotherapists. The correlation coefficients between these factors and the indicators of general and primary morbidity of mental disorders are (in absolute value) in the range of 0.65-0.93 with a reliability level of 95%. At the second stage, linear regression (one-factor, two-factor) and nonlinear (logistic) models were obtained, linking the incidence of mental disorders with the selected factors. The obtained regression models are characterized by high reliability with a coefficient of determination R^2^ in the range 0.81-0.92 for single-factor and, respectively, 0.90-0.98 for two-factor models. The obtained nonlinear logistic model for the indicator of general morbidity allowed us to obtain a maximum morbidity value for the Russian population equal to 34.4% of the population. This result practically coincides with the result obtained in the well-known work [2]. In this epidemiological study on the European continent, it was found that 38.2% of European residents suffer from mental disorders.

**Conclusions:**

The obtained models make it possible to quickly monitor the impact of medical, demographic, socio-economic factors and changes in the personnel resource of psychiatric care on the morbidity rates of the Russian population. 1. Mitikhin V., Yastrebov V. et al. *Neuroscience and Behavioral Physiology.* 2019; 49(2): 233-239. doi: 10.1007/s11055-019-00720-4. 2. Wittchen, H. U., Jacobi, F., Rehm, J., et al. *European Neuropsychopharmacology.* 2011; 21: 655-679. doi:10.1016/j.euroneuro.2011.07.018

**Disclosure of Interest:**

None Declared

